# Magnon mode transition in real space

**DOI:** 10.1038/s41598-022-22555-9

**Published:** 2022-12-07

**Authors:** Kazuki Iida, Katsuaki Kodama, Yasuhiro Inamura, Mitsutaka Nakamura, Lieh-Jeng Chang, Shin-ichi Shamoto

**Affiliations:** 1 Research Center for Neutron Science and Technology, Comprehensive Research Organization for Science and Society (CROSS), Tokai, Ibaraki 319-1106 Japan; 2 Materials Sciences Research Center, Japan Atomic Energy Agency, Tokai, Ibaraki 319-1195 Japan; 3 J-PARC Center, Japan Atomic Energy Agency, 2-4 Shirakata, Tokai, Naka, Ibaraki 319-1195 Japan; 4 Department of Physics, National Cheng Kung University, Tainan, 701 Taiwan; 5 Institute of Physics, Academia Sinica, Taipei, 115201 Taiwan; 6 Advanced Science Research Center, Japan Atomic Energy Agency, 2-4 Shirakata, Tokai, Naka, Ibaraki 319-1195 Japan; 7 Advanced Meson Science Laboratory, RIKEN, Wako, Saitama 351-0198 Japan

**Keywords:** Materials science, Nanoscience and technology, Physics

## Abstract

Spin excitation of an ilmenite FeTiO_3_ powder sample is measured by time-of-flight inelastic neutron scattering. The dynamic magnetic pair-density function *D*_*M*_(*r, E*) is obtained from the dynamic magnetic structure factor *S*_*M*_(*Q, E*) by the Fourier transformation. The real space spin dynamics exhibit magnon mode transitions in the spin–spin correlation with increasing energy from no-phase-shift to π-phase-shift. The mode transition is well reproduced by a simulation using the reciprocal space magnon dispersions. This analysis provides a novel opportunity to study the local spin dynamics of various magnetic systems.

## Introduction

Reciprocal expression has been traditionally used for the spin excitations as a magnon dispersion, which can be measured directly by inelastic neutron scattering (INS) with the energy and the momentum transfers in various magnets. The magnon (spin wave) dispersion has been a unique description of the elementary excitation. Meanwhile, recent developments of INS spectrometers at pulsed neutron sources provide extremely high signal to noise ratio above a few hundreds. This low background enables us to measure the weak magnetic diffuse scattering in a wide *Q*-range. So far, total scattering spectrometers in the facilities have been used for local structure analysis that utilizes a wide *Q*-range of the scattering pattern by the time-of-flight (TOF) method in various liquid and amorphous materials, and even crystalline solids^1^. Recently, the research has been extended to the inelastic scattering measurement of the dynamic pair-density function^2^. The time-dependence has also been discussed by Fourier transform on the energy axis as well. Meanwhile, the magnetic pair-density function analysis has also been applied to several materials such as a spin-glass system^3^. One of the pioneering works is the spin–spin correlation study on an amorphous alloy (Mn_0.4_Ni_0.6_)_75_P_16_B_6_Al_3_,^4^ where polarized neutron scattering has been carried out using the scattering pattern up to *Q*_max_ = 7 Å^-1^. The magnetic pair distribution function analysis is formulated in Ref.^5^. The spin density background is evaluated in Ref.^6^. As for the dynamics, the dynamic atomic pair-density function analysis has been performed in amorphous solids like SiO_2_ glass^7,8^. The method is also applied to a ferroelectric material^9^. After these pioneering works, the method has been widely used in liquid and glassy materials^2^.

**Figure 1:**
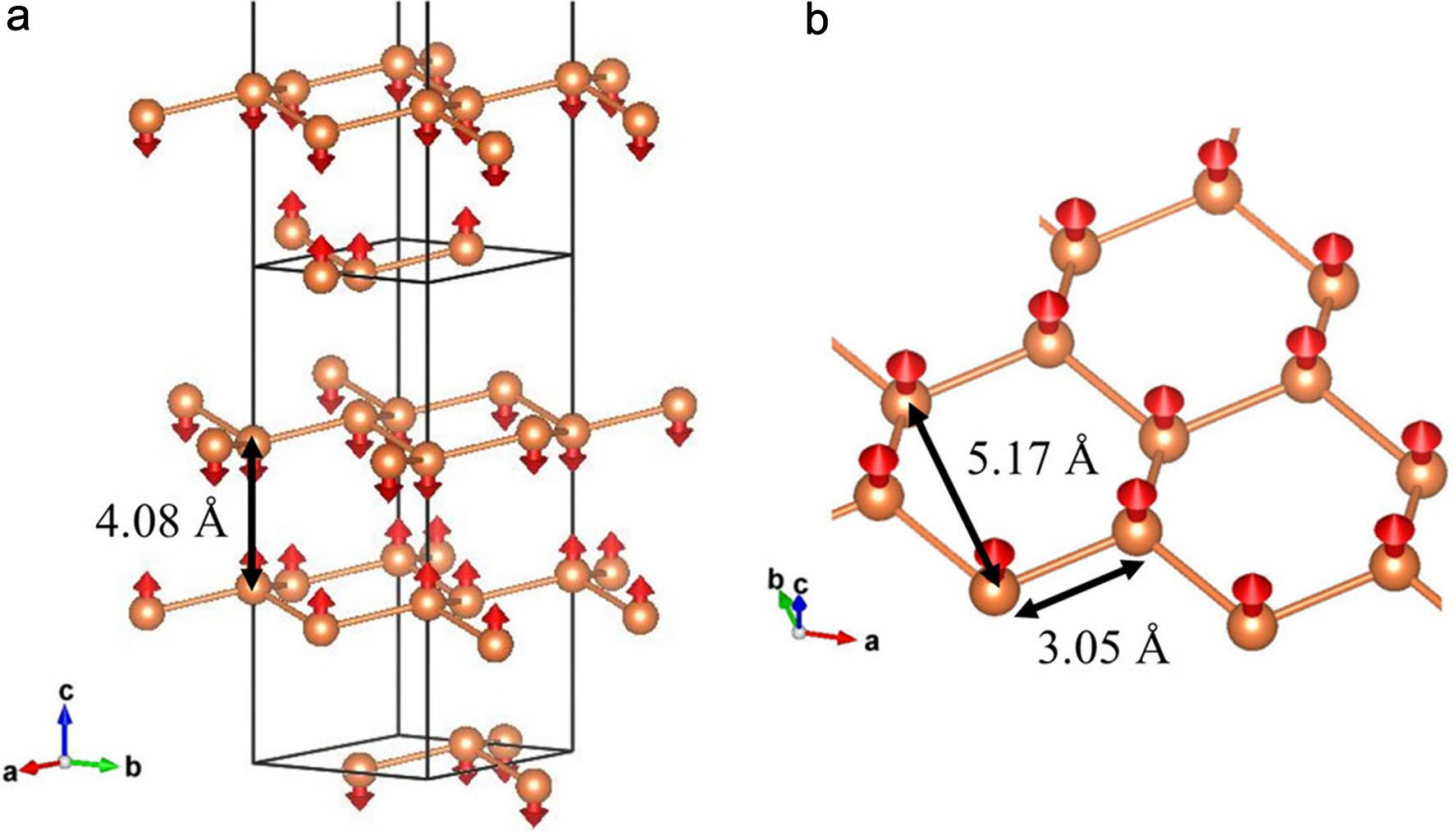
Magnetic structure of FeTiO_3_. Every other Fe atom (brown ball) slightly buckles along the *c*-axis. The spin (red arrow) directs along the *c*-axis with a small tilting of about 2°^10^. The nearest-neighbor bonds (brown rods) between Fe spins with *r* = 3.05 Å form a honeycomb lattice in the *ab*-plane.

Here, we measured the magnetic excitation of a powder sample of FeTiO_3_ by TOF INS. The crystal and magnetic structures of FeTiO_3_ are depicted in Fig. 1. FeTiO_3_ powder sample was selected because the magnon dispersions with optical modes are well studied^10^ and the Fe^2+^ magnetic moment is relatively large. The dynamic magnetic structure factor was analyzed by the dynamic magnetic pair-density function (DymPDF) analysis. The DymPDF analysis program is implemented in the ‘Utsusemi’ visualization software^11^.

### Dynamic magnetic pair-density function analysis

The dynamic pair-density function of the lattice *D*_*L*_(*r, E*) is calculated based on the following equation^7,8^.

1 $${D}_{L}\left(r,E\right)=\frac{2}{\pi }\int Q\left[\frac{{S}_{L}\left(Q,E\right)-{S}_{s0}\left(Q,E\right)}{B{Q}^{2}\mathrm{exp}\left(-{Q}^{2}\langle {u}^{2}\rangle /3\right)\frac{\langle n\left(E\right)+1\rangle }{E}}\right]\mathrm{sin}(Qr)dQ$$

where *S*_*L*_(*Q, E*) is the lattice part of the dynamic structure factor at the scattering vector *Q* and the energy *E*, and the self-dynamic structure factor *S*_*s0*_(*Q, E*) can be written as follows.

2 $${S}_{s0}\left(Q,E\right)=B{Q}^{2}exp\left(-{Q}^{2}\langle {u}^{2}\rangle /3\right)\frac{\langle n\left(E\right)+1\rangle }{E}$$

where *B* is a constant; <*u*^2^> is the mean-square atomic displacement; *n*(*E*) is the Bose factor at an energy *E*.

**Figure 2:**
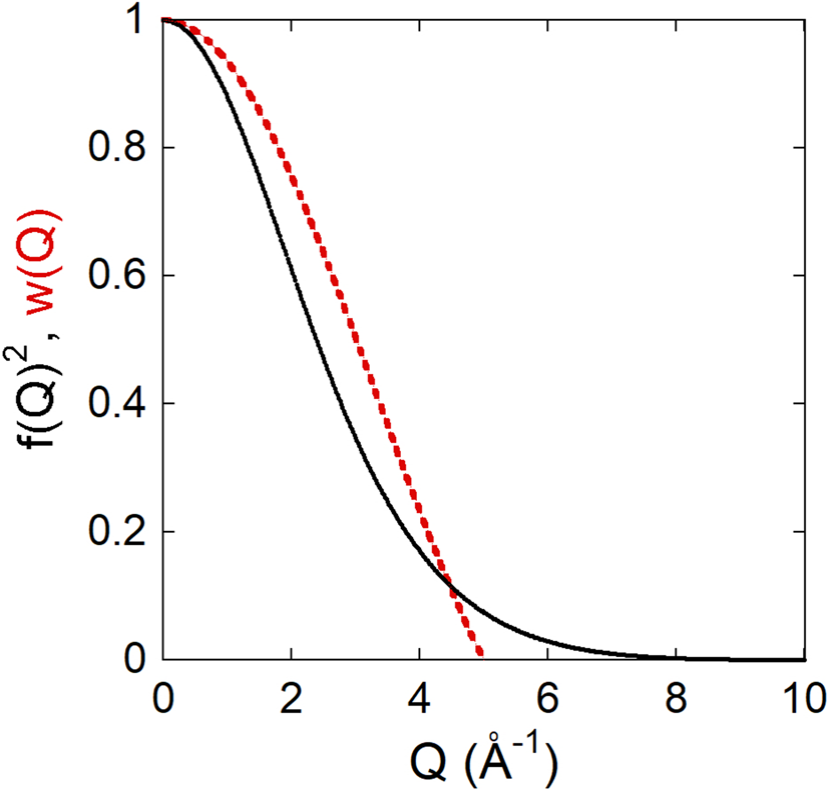
Squared magnetic form factor of Fe^2+^ as a function of *Q* (solid black line) and window function of Eq. (5) with *Q*_max_ = 5 Å^−1^ (broken red line).

In Eq. (1), *D*_*L*_(*r, E*) is multiplied by *E* based on the energy dependence of the phonon intensity. In the present case of magnetic excitation, we can omit the energy dependence in the following Eq. (3). In contrast to the phonon, the magnetic signals sharply decrease with increasing the scattering vector *Q* by the squared magnetic form factor *f*_M_(*Q*)^2^. Because of the effect, the magnetic signals become negligibly small above *Q* = 5 Å^−1^ for 3*d* transition metals such as Fe^2+^, as shown in Fig. 2. Therefore, the maximum *Q* is effectively limited to about 5 Å^−1^ for the DymPDF analysis, resulting in the limitation of the *r*-resolution. In other words, the outer orbital with magnetic moments such as 3*d* spins spread to some extent. Because of this reason, the maximum *Q* was set to be 4.3–5 Å^−1^ in the present DymPDF analysis. This small *Q*_max_ results in the suppression of the phonon contribution in the analysis.

The dynamic magnetic structure factor *S*_*M*_(*Q, E*) of FeTiO_3_ was Fourier transformed to the dynamic magnetic pair-density function *D*_*M*_(*r, E*) based on the following Eq. (3), after being divided by Bose factor *n*(*E*) + 1 and squared magnetic form factor *f*_M_(*Q*)^2^ of Fe^2+^.

3 $${D}_{M}\left(r,E\right)=\frac{2}{\pi }{\int }_{{Q}_{min}}^{{Q}_{max}}Q\left[\frac{{S}_{M}\left(Q,E\right)}{\langle n\left(E\right)+1\rangle }-1\right]w\left(Q\right)\mathrm{sin}(Qr)dQ$$

where *S*_*M*_(*Q, E*) is obtained as follows.

4 $$\frac{{S}_{M}\left(Q, E\right)}{\langle n\left(E\right)+1\rangle }-1=\frac{S\left(Q, E\right)-{S}_{s}\left(Q, E\right)-{S}_{L}\left(Q, E\right)-{S}_{0}\left({Q}_{max}, E\right)}{{f}_{M}^{2}\left(Q\right)\langle n\left(E\right)+1\rangle }$$

The window function *w*(*Q*) is written as,

5 $$w\left(Q\right)=\frac{{Q}_{max}}{\pi Q}\mathrm{sin}\left(\frac{\pi Q}{{Q}_{max}}\right)$$

This window function reduces the error by suppressing the difference of *S*_*M*_(*Q, E*)/<*n*(*E*) + 1> from unity near *Q*_max_. The window function effect can be found in Suppl. Note 1. Figure 2 shows an example of the window functions with *Q*_max_ = 5 Å^−1^.

Here, the incoherent dynamic structure factor *S*_*S*_(*Q, E*) and the phonon dynamic structure factor *S*_*L*_(*Q, E*) in Eq. (4) are approximated into one *Q*^2^ function as follows.

6 $${S}_{S}\left(Q, E\right)+{S}_{L} \left(Q, E\right) \sim A{\left(Q-{Q}_{max}\right)}^{2}$$

where *A* is a constant parameter obtained by a least-squares fitting to minimize the integral of [*S*(*Q, E*) − *A*(*Q* − *Q*_max_)^2^ − *S*_0_(*Q*_max_, *E*)] from *Q*_min_ to *Q*_max_. This subtraction corrects the oscillation balance of *S*_*M*_(*Q, E*)/<*n*(*E*) + 1 > around unity, leading to *D*_*M*_(0*, E*)/<*n*(*E*) + 1 > = 0. The *S*_0_(*Q*_max_, *E*) in Eq. (4) is determined from the following Eq. (7) to converge the *D*_*M*_(*r, E*).

7 $$S\left({Q}_{max}, E\right)-{S}_{s}\left({Q}_{max}, E\right)-{S}_{L}\left({Q}_{max}, E\right)-{S}_{0}\left({Q}_{max}, E\right)=0$$

The combination of Eqs. (5–7) protects the divergence of the dynamic magnetic pair-density function *D*_*M*_(*r, E*). Equation (6) corresponds to second-degree polynomial correction in the PDF analysis^12^. The detailed processes are described in Suppl. Note 2.

### Local spin dynamics of FeTiO_3_ in real space

**Figure 3:**
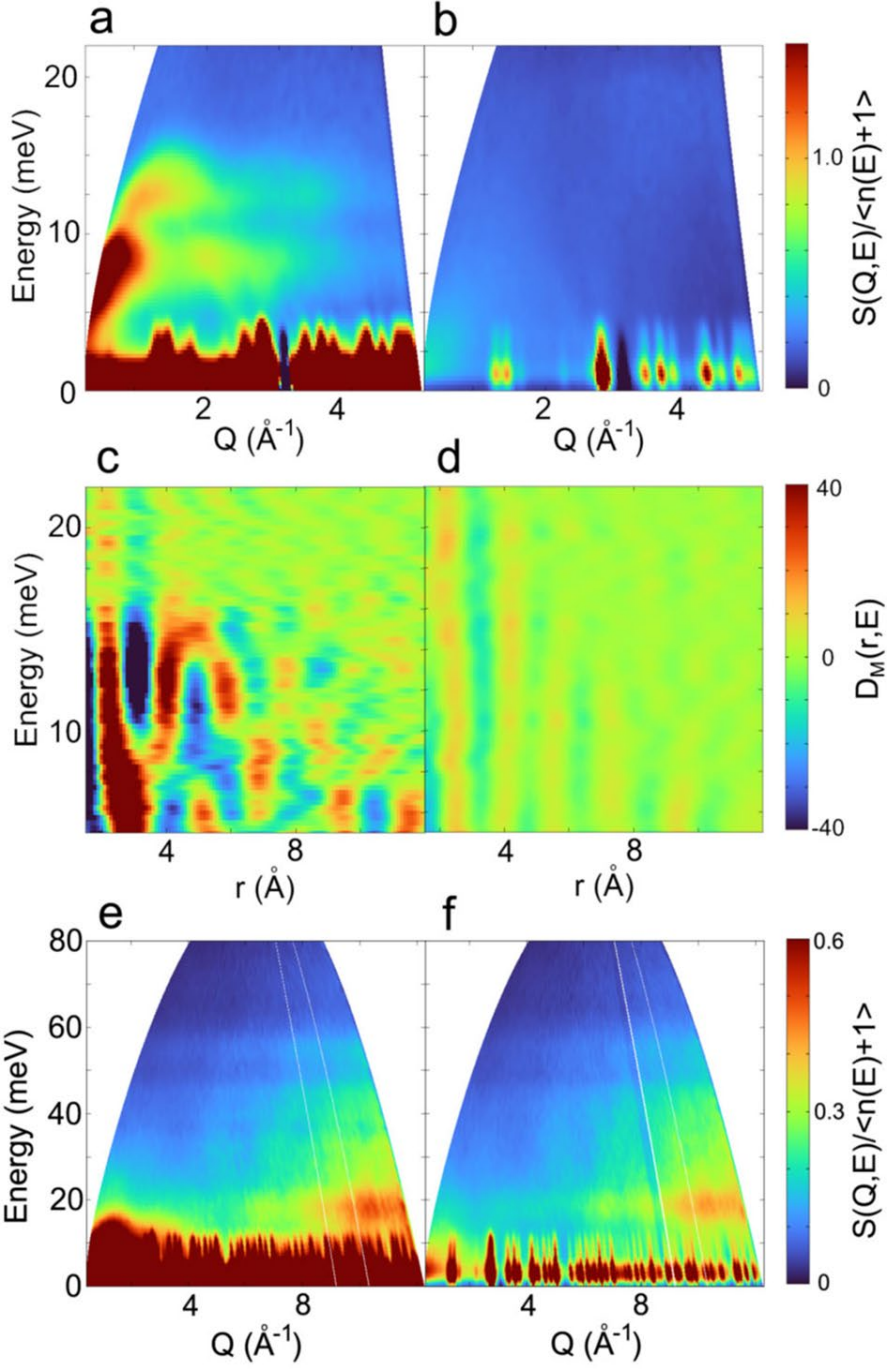
**(a)** Bose-factor corrected dynamic structure factor *S*(*Q, E*)/<*n*(*E*) + 1 > of FeTiO_3_ measured with *E*_i_ = 46 meV at *T* = 8 K. (**b)** at *T* = 200 K. (**c)** Dynamic magnetic pair-density function *D*_*M*_(*r, E*) of FeTiO_3_ at *T* = 8 K. (**d)** at *T* = 200 K. (**e)** Bose-factor corrected dynamic structure factor *S*(*Q, E*)/<*n*(*E*) + 1 > of FeTiO_3_ measured with *E*_i_ = 95 meV at *T* = 8 K. (**f)** at *T* = 200 K. Low-energy spiky peaks in *S*(*Q, E*)/<*n*(*E*) + 1 > are originated from strong Bragg peaks, which extend with the energy-resolution.

Bose-factor corrected dynamic structure factors *S*(*Q, E*)/<*n*(*E*) + 1 > of FeTiO_3_ measured at *T* = 8 and 200 K with *E*_i_ = 46 and 95 meV are shown in Fig. 3. The *Q–E* range of *S*(*Q, E*) with *E*_i_ = 46 meV was limited for the analysis up to 5 Å^−1^ and 22 meV in Fig. 3a,b, where only magnetic signals are visible. The spin excitation was clearly observed at 8 K below 3 Å^−1^ and 16 meV in Fig. 3a. A gap-like feature is observed at *E* = 11 meV, which can be attributed to the zone boundary gap between 9 and 12 meV at (0.5, 0, 0)^10,13^. The *Q–E* range of *S*(*Q, E*) in the case of *E*_i_ = 95 meV spreads up to 12 Å^−1^ and 80 meV (Fig. 3e,f). However, the magnon excitations are not well-defined due to the low resolution. Instead, phonon excitations become visible above 5 Å^−1^ and up to about 60 meV. The results support our setting of *Q*_max_ < 5 Å^−1^ for the DymPDF analysis.

As shown in Fig. 1, Fe^2+^ (3*d*^6^, *S* = 2) spins in FeTiO_3_ order ferromagnetically within the honeycomb planes, which are then stacked antiferromagnetically along the *c*-axis. As all spins in a single honeycomb plane are oriented along the same direction, their fluctuations in phase with each other will appear as positive peaks in the DymPDF. We thus expect positive peaks at *r* = 3.0 Å and = 5.1 Å which are the nearest-neighbor and the next-nearest-neighbor Fe^2+^–Fe^2+^ bond distances within honeycomb planes, respectively. In the meanwhile, the second shortest Fe^2+^–Fe^2+^ bond in FeTiO_3_ is at *r* = 4.0 Å which is vertically connecting two adjacent honeycomb planes. As the interplanar spin coupling is antiferromagnetic, the low-energy fluctuations at this distance will appear as negative peaks in the DymPDF. The low-energy parts in Fig. 3c indeed show such peaks with expected sign changes between *r* = 3.0 Å and 5.1 Å. Figure 3d shows, however, that these dynamic spin–spin correlations of the ordered magnet at *T* = 8 K apparently are absent at *T* = 200 K well above the Néel temperature. Nevertheless, we notice weak pair-density correlations remaining in the paramagnetic phase at 200 K (Fig. 3d) at the same positions, suggesting dynamic atomic correlations via phonon vibrations. The positive phonon peak at *r* = 2.1–2.2 Å above 16 meV can be attributed to the Fe–O bonds, whereas the negative phonon peak at *r* = 3 Å above 16 meV may be the Fe–Ti bonds due to the scattering length sign change between Fe and Ti.

**Figure 4:**
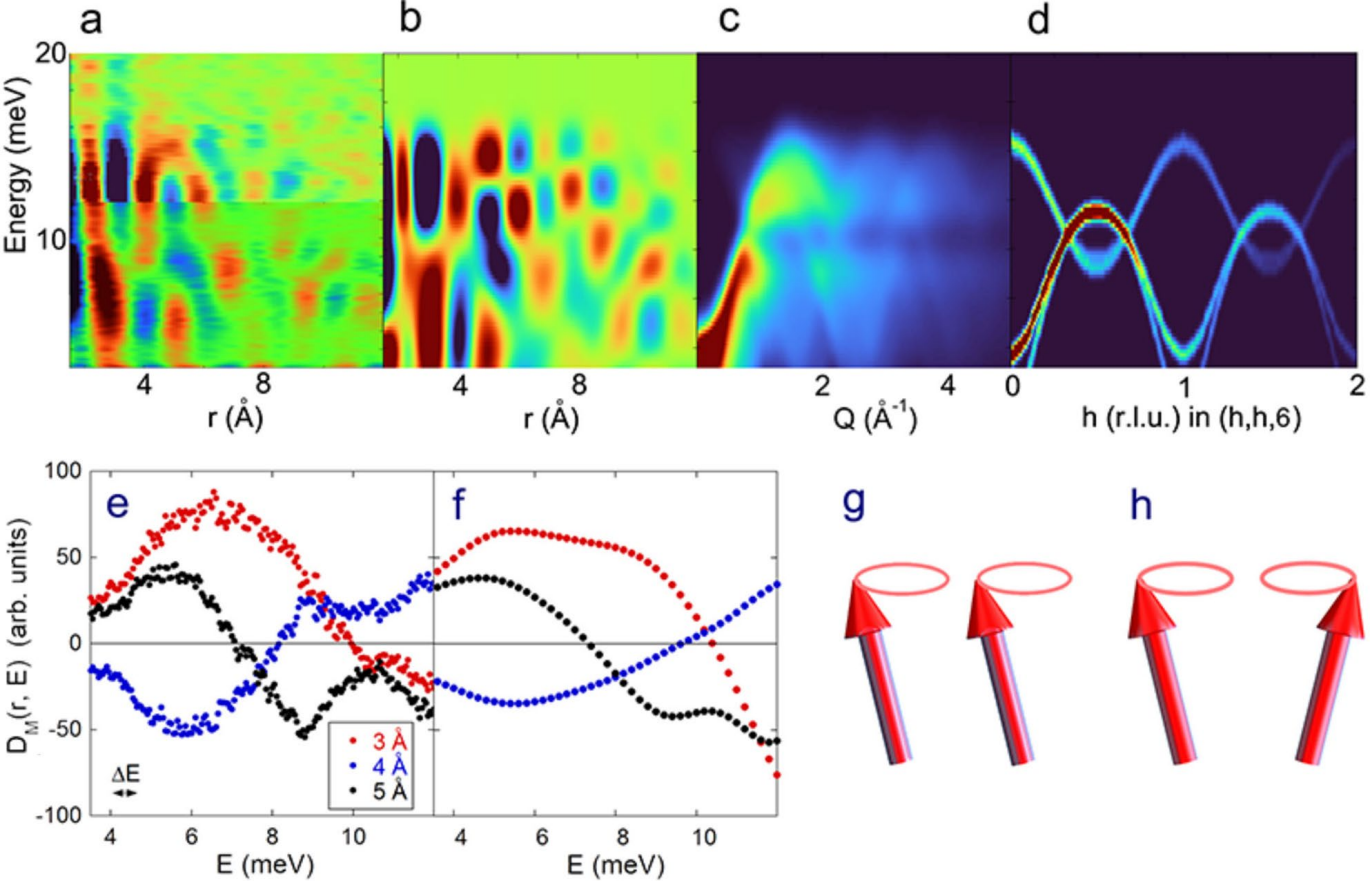
**(a)** Combined DymPDF *D*_*M*_(*r, E*) pattern at *T* = 8 K measured with *E*_i_ = 18 (*E* < 12 meV) and 46 meV (*E* > 12 meV). The magnon mode transition at *r* = 3 Å is observed at about 10 meV. (**b)** Simulated *D*_*M*_^*cal*^(*r, E*) pattern based on the SpinW powder averaged magnon dispersions^14^. (**c)** Powder averaged *S*_*M*_^*cal*^(*Q, E*)/<*n*(*E*) + 1 > calculated by SpinW based on the exchange parameters in Ref.^10^. (**d)** Magnon dispersions calculated by SpinW. (**e)** Energy dependence of *D*_*M*_(*r, E*) at *r* = 3, 4, and 5 Å integrated with *r*-width of 1 Å (*E*_i_ = 18 meV). The energy-resolution Δ*E* is shown on the left-side bottom. **f** Energy dependence of *D*_*M*_^*cal*^(*r, E*) calculated by SpinW at *r* = 3, 4, and 5 Å. (**g)** Schematic magnon mode with no-phase-shift between the nearest-neighbor spins. (**h)** Schematic magnon mode with π-phase-shift between the nearest-neighbor spins.

Figure 4 shows the comparison between the present *D*_*M*_(*r, E*) and the simulated *D*_*M*_^*cal*^(*r, E*)/<*n*(*E*) + 1 > based on the exchange parameters reported in Ref.^10^. Figure 4a shows a wide-energy DymPDF *D*_*M*_(*r, E*) pattern combined from two data sets measured with *E*_i_ = 18 (*E* < 12 meV) and 46 meV (*E* > 12 meV). The magnon mode transition at *r* = 3 Å is observed at about 10 meV. The powder averaged *S*_*M*_^*cal*^(*r, E*)/<*n*(*E*) + 1 > pattern calculated by SpinW^14^ is shown in Fig. 4c. The calculated original magnon dispersions are shown in Fig. 4d. As expected, the present *D*_*M*_(*r, E*) (Fig. 4a) is reasonably consistent with the simulated *D*_*M*_^*cal*^(*r, E*) (Fig. 4b). The energy-dependences of *D*_*M*_(*r, E*) at *r* = 3, 4, and 5 Å are shown in Fig. 4e, which roughly correspond to the first, second, and third nearest-neighbor bond lengths, respectively. Interestingly, they exhibit sign changes around 10, 8, and 7 meV, respectively, suggesting the reversal of the spin–spin correlations. These sign changes can be interpreted as the changes in relative phases as the magnon dispersions approach the Brillouin zone boundary. At *r* = 3 Å, for instance, the low-energy part accounts for the cooperatively oscillating acoustic mode without a phase difference between a pair of nearest-neighbor spins as illustrated in Fig. 4g. Meanwhile, the sign change around 10 meV can be interpreted as the magnon mode attaining π-phase difference illustrated in Fig. 4h. This sign change with increasing energy reminds us of the Ni phonon mode change from acoustic to optical modes observed by DyPDF^9^. Naively, the transition energy of 10 meV is expected to correspond to the dispersion change from acoustic to optical magnon dispersions in Fig. 4d. The highest energy of the acoustic mode reaches 12 meV, whereas the lowest energy of the optical mode extends to 7 meV. The 10 meV is near the center of the overlapping modes. Each nearest neighbor bond length may correspond to a typical energy in the energy range from 7 to 12 meV. However, the present DymPDF represents local magnon mode different from the magnon dispersions in Fig. 4d. In a real space image, even the acoustic mode can show π-phase-shift at the zone boundary in the acoustic magnon dispersion. Therefore, the magnon mode transition does not simply correspond to the dispersion change from acoustic to optical ones. Anyway, the real space profile was well reproduced by the spin-wave dispersions, suggesting a close correlation between them.

In addition, there are some noticeable anomalies in Fig. 4e. The first kink appears as a peak or a bottom at 5.5–6.5 meV for these bond lengths. The second kink is at about 9 meV. The third kink is at about 10.5 meV clearly observed for all the bonds. The first kink energy agrees with the gap of one acoustic magnon mode. The second kink energy corresponds to the bottom energy of the optical magnon mode. The third kink energy is the crossing point of the acoustic and optical modes. The SpinW simulation roughly reproduced these kinks as shown in Fig. 4f. The reason why these curves do not match exactly with the simulation requires further study. However, this method provides a novel opportunity, for example, to observe the local spin dynamics of magnetic nanoclusters, which, so far, had been difficult to study.

In summary, we successfully observed the local magnon mode change on FeTiO_3_ in real space by the dynamic magnetic pair-density function (DymPDF) analysis. This real space image provides a novel possibility to study local spin dynamics in addition to the nearly static magnetic pair-density of various magnetic systems at low energy. The latter is particularly important because nuclear scattering components can be naturally removed by limiting *Q*-range. It means that a tedious process to subtract nuclear Bragg peaks from the measured pattern can be avoided. Possible suitable research by DymPDF would be molecular cluster magnets, nanomagnets, local magnetic clusters, and magnetic short-range orders in addition to magnetic amorphous alloys and magnetic quasicrystals. The local magnetic clusters are known to appear in spin frustration systems as a spin molecule in MgCr_2_O_4_^15^. In a superconducting state, spin resonance mode appears in an unconventional superconductor such as CeCoIn_5_^16^. By DymPDF analysis, it becomes possible to check whether spin-singlet or spin-triplet state is realized in the superconducting state, in addition to the correlation length that can be compared with the superconducting coherence length. Magnetic percolation network such as fracton^17^ or Mott transition by anion exchange^18^ may also be a suitable system to study a decay of the magnetic pair-density as a function of a bond length in the network. DymPDF analysis can also be used to study the spin dynamics of a crystalline magnet that can be synthesized only in powder form. The application is not limited to these systems. There can be vast other applications by the DymPDF analysis.

## Methods

A FeTiO_3_ powder sample of 99.5% purity was purchased from Kojundo Chemical Lab. Co., Ltd. The X-ray diffraction powder pattern did not include any impurity phase. The magnetic structure is shown in Fig. 1. The space group is *R*-3 (#148) with lattice parameters of *a* = 5.087 Å and *c* = 14.092 Å (Hexagonal)^19^. FeTiO_3_ exhibits an antiferromagnetic phase transition at *T*_N_ = 58.0 K^19^. The powder with a weight of 5.58 g was put into an aluminum cell. Inelastic neutron scattering measurements were carried out at the chopper spectrometer 4SEASONS with a multi-*E*_i_ option in J-PARC with a proton beam power of 600 kW^20^. The used incident energies were 17.8, 46.0, and 94.7 meV under a Fermi chopper frequency of 300 Hz. The energy resolutions at *E* = 0 for *E*_i_ = 17.8, 46.0, and 94.7 meV are 0.67, 2.48, and 7.43 meV, respectively. The dynamic structure factor *S*(*Q, E*) was subtracted by empty aluminum cell data measured under the same condition. The detector efficiency depending on *E*_f_ was corrected in the ‘Utsusemi’ software^11^. Crystal and magnetic structures of FeTiO_3_ were drawn by the software ‘VESTA’^21^. The magnon dispersions in Fig. 4c,d are calculated by ‘SpinW’ software based on the linear spin wave theory with the Holstein–Primakoff approximation^14^.

## Supplementary Information

Supplementary Information.

## Data Availability

The datasets used and analyzed during the current study available from the corresponding author upon reasonable request.
