# Supplementary material for: Magnon mode transition in real space
Source: Sci Rep. 2022 Dec 7;12:20663. doi: 10.1038/s41598-022-22555-9 (PMC9729307; doi:10.1038/s41598-022-22555-9)
Supplement: Supplementary file 1 — Supplementary Information. [file 41598_2022_22555_MOESM1_ESM.docx]

**Supplementary Materials:**

**Magnon mode transition in real space**

Kazuki Iida^a^, Katsuaki Kodama^b^, Yasuhiro Inamura^c^, Mitsutaka Nakamura^c^, Lieh-Jeng Chang^d^, Shin-ichi Shamoto^a,d,e,f,^*

^a^*Research Center for Neutron Science and Technology,*

*Comprehensive Research Organization for Science and Society (CROSS),*

*Tokai, Ibaraki 319-1106, Japan*

^b^*Materials Sciences Research Center, Japan Atomic Energy Agency, Tokai,*

*Ibaraki 319-1195, Japan*

^c^*J-PARC center, Japan Atomic Energy Agency, 2-4 Shirakata, Tokai, Naka, Ibaraki 319-1195, Japan*

^d^*Department of Physics, National Cheng Kung University, Tainan 701, Taiwan*

^e^*Advanced Science Research Center, Japan Atomic Energy Agency, 2-4 Shirakata, Tokai, Naka, Ibaraki 319-1195, Japan*

^f^*Advanced Meson Science Laboratory, RIKEN, Wako, Saitama 351-0198, Japan*

*s_shamoto@cross.or.jp

**Supplementary Note 1: Magnon and Phonon Components in a Dynamic Structure Factor of FeTiO_3_ and Effect of Window Function for DymPDF**

In the current data calculation process of the dynamic magnetic pair-density function (DymPDF), it is essential to estimate how much the magnon and the phonon contribute to the dynamic structure factor of *S*(*Q, E*)/<*n*(*E*)+1> of FeTiO_3_. To check their contributions, a wide-*Q* range of data is suitable because of their opposite *Q*-dependences. Because of the squared magnetic form factor of Fe^2+^, the magnon part is expected to be negligible above *Q*= 5 Å^-1^. On the other hand, the phonon intensity increases in proportional to *Q*^2^. A thermal factor *B* is often included in the *Q*-dependence of the intensity. However, the *B* was negligibly small in Suppl. Fig. 1a. The phonon intensity was estimated by fitting the intensity with a simple equation of *C*_0_+*C*_1_ *Q*^2^ above 5.5 Å^-1^. The constant term *C*_0_ was attributed to the multiple scattering. The remained part above 5.5 Å^-1^ is shown as the phonon component in Suppl. Fig. 1a. After the phonon and the constant terms are subtracted from the dynamic structure factor of *S*(*Q, E*)/<*n*(*E*)+1>, the magnon component remains below 6 Å^-1^ as shown in Suppl. Fig. 1a. Although a certain amount of phonon component remains in the dynamic structure factor of *S*(*Q, E*)/<*n*(*E*)+1>, the intensity below 5 Å^-1^ was attributed to the magnon component here. Note that the magnon intensity at *E*= 15 meV is weak, as shown in the inset of Suppl. Fig. 2a. Hence, our approximation can be justified for this FeTiO_3_ analysis. The lower energies than 15 meV were avoided because of the intensity contamination from the elastic scattering due to the coarse energy resolution at *E_i_*= 95 meV. At *T*= 200 K, the intensity ratio of the phonon component to the magnon one becomes about four times larger than that at 8 K. Hence, it is inevitable that the phonon effect appears even below 5 Å^-1^ in the DymPDF at *T*= 200 K.

The window function effects are shown as examples of the dynamic magnetic structure factor *Q*[*S_M_*(*Q, E*)/<*n*(*E*)+1>-1] and the dynamic magnetic pair-density function *D_M_*(*r, E*) in the Suppl. Figs. 1b and c, respectively. Without the window function, the *Q*[*S_M_*(*Q, E*)/<*n*(*E*)+1>-1] becomes largely scattered with large error bars in the high-*Q* region. The large erroneous values result in the unphysical oscillation of *D_M_*(*r, E*) as shown in Suppl. Fig. 1c.

In summary, our approximation neglecting the phonon component can be justified for FeTiO_3_. This is partly due to the large magnetic moment of Fe^2+^ (~ 4 μ_B_). As for the phonon intensity, elements of Fe and O have relatively large scattering lengths. But Ti has a negative scattering length, which may cancel the other scattering lengths for some phonon modes. In total, the phonon intensity may not be strong for FeTiO_3_ especially at low energy phonon modes. In general, the ratio of scattering intensities of the magnon to the phonon varies depending on the sample material. Hence, special attention must be paid on the ratio for this DymPDF analysis by checking the intensities as shown in Suppl. Fig. 1a.

Supplementary Figure 1: **a** Wide *Q*-range of dynamic structure factor *S*(*Q, E*)/<*n*(*E*)+1> of FeTiO_3_ at *E* = 15 meV (1 meV E-width) observed at *T*= 8 K for *E*_i_= 95 meV. Phonon component was estimated based on the *Q*^2^ fitting in the high-*Q* range, whereas magnon component was the rest of the *S*(*Q, E*)/<*n*(*E*)+1> after subtracting the phonon component and a constant *C*_0_ term. **b** Dynamic magnetic structure factor multiplied by *Q*, *Q*[*S_M_*(*Q, E*)/<*n*(*E*)+1>-1] in Eq. (4). Red closed circles are without a window function *w*(*Q*) of Eq. (5), while black closed circles are multiplied by the window function. **c** Dynamic magnetic pair-density function, *D_M_*(*r, E*). Red closed circles are calculated without a window function, while black closed circles are with the window function. The window function effect appears clearly.

**Supplementary Note 2: Data Calculation Process of Dynamic Magnetic Pair-density Function**

The data calculation process of the dynamic magnetic pair-density function (DymPDF) is described in this note. Here, for simplicity, one DymPDF example of FeTiO_3_ sliced at *E*= 6 meV (1 meV energy width, *E*_i_ = 46 meV, *T* = 8 and 200 K) is shown in detail. The observed dynamic structure factor *S*(*Q, E*) by dividing the Bose factor <*n*(*E*)+1> (*E*_i_ = 46 meV, *T* = 8 K) is shown in the inset of Suppl. Fig. 2a. The dynamic structure factor *S*(*Q, E*)/<*n*(*E*)+1> at 6 meV is shown as a function of **Q** in Suppl. Fig. 2a. Based on this *Q*-dependent pattern, the data calculation starts. The incoherent dynamic structure factor *S_S_*(*Q, E*) and the phonon dynamic structure factor *S_L_*(*Q, E*) in Eq. (4) are approximated into one *Q*^2^ function in Eq. (6). The constant parameter *A* for Suppl. Fig. 2a was determined to be 0.05628 by a least-squares fitting to minimize the integral of [*S*(*Q, E*)-*A*(*Q*-*Q*_max_)^2^-*S*_0_(*Q*_max_, *E*)] from *Q*_min_ to *Q*_max_. The *S*_0_(*Q*_max_, *E*) in Eq. (4) was determined from Eq. (7) to converge the *D_M_*(*r, E*). After the subtraction, it is divided by squared magnetic form factor *f_M_*^2^(*Q*). The obtained dynamic magnetic structure factor *S_M_*(*Q, E*)/ <*n*(*E*)+1>-1 is shown in Suppl. Fig. 2b. It was Fourier transformed by using Eq. (3). The calculated DymPDF is shown in Suppl. Fig. 2c. This calculation process was carried out one by one at each energy-sliced pattern, resulting in Fig. 3c. The same process was carried out for the dynamic structure factor *S*(*Q, E*) observed at *T*= 200 K. The results are shown in Suppl. Fig. 3.


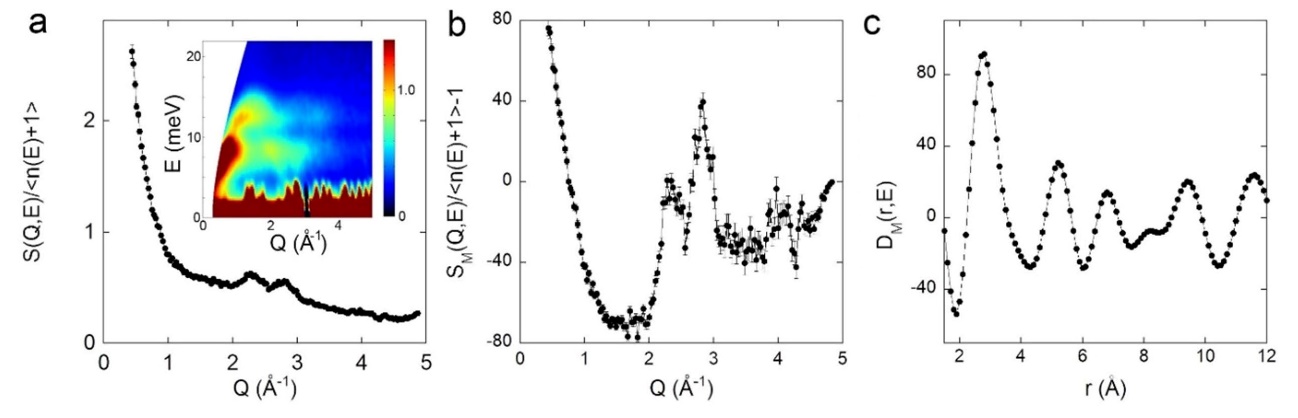


Supplementary Figure 2: **a** Dynamic structure factor *S*(*Q, E*)/<*n*(*E*)+1> of FeTiO_3_ of *E* = 6 meV observed at *T*= 8 K. Inset shows the *Q*-*E* map of the dynamic structure factor *S*(*Q, E*)/<*n*(*E*)+1>. **b** Obtained dynamic magnetic structure factor *S_M_*(*Q, E*)/<*n*(*E*)+1>-1 of *E* = 6 meV at *T*= 8 K. c Dynamic magnetic pair-density function *D_M_*(*r, E*) of *E* = 6 meV at *T*= 8 K. Every intensity is an arbitrary unit.

Supplementary Figure 3: **a** Dynamic structure factor *S*(*Q, E*)/<*n*(*E*)+1> of FeTiO_3_ of *E* = 6 meV observed at *T*= 200 K. Inset shows the *Q*-*E* map of the dynamic structure factor *S*(*Q, E*)/<*n*(*E*)+1>. **b** Obtained dynamic magnetic structure factor *S_M_*(*Q, E*)/<*n*(*E*)+1>-1 of *E* = 6 meV at *T*= 200 K. c Dynamic magnetic pair-density function *D_M_*(*r, E*) of *E* = 6 meV at *T*= 200 K. Every intensity is an arbitrary unit.

The dynamic structure factors *S*(*Q, E*)/<*n*(*E*)+1> below *Q* = 5 Å^-1^ in Suppl. Fig. 2 were **regarded to consist only of magnetic scattering components** because there was no phonon excitation. The Fourier transformation of the dynamic magnetic structure factor results in the dynamic magnetic pair-density function *D_M_*(*r, E*) as shown in Suppl. Fig. 2c based on Eq. (1). In the case of 200 K, the intensity of the dynamic structure factor *S*(*Q, E*)/<*n*(*E*)+1> was weak (Suppl. Fig. 3a). Nevertheless, the calculated DymPDF pattern above 16 meV at 200 K shows some peaks which look pretty similar to that at 8 K. In addition to this similarity, the first positive peak position at *r* = 2.1-2.2 Å above 16 meV matches the Fe-O bond distance of 2.08 and 2.20 Å, whereas the negative phonon peak at *r* = 3 Å above 16 meV may correspond to the Fe-Ti bond length of 2.94 Å due to the scattering length sign change between Fe and Ti. However, the first positive peak increases the bond distance with decreasing energy. These weak peaks must be carefully discussed because of the possible large errors. Their error estimations are still underway.
